# Acetylsalicylic acid supplementation improves protein utilization efficiency while vitamin E supplementation reduces markers of the inflammatory response in weaned pigs challenged with enterotoxigenic *E. coli*

**DOI:** 10.1186/s40104-016-0118-4

**Published:** 2016-10-03

**Authors:** Jae Cheol Kim, Bruce P. Mullan, John L. Black, Robert J. E. Hewitt, Robert J. van Barneveld, John R. Pluske

**Affiliations:** 1 School of Veterinary and Life Sciences, Murdoch University, Murdoch, WA 6150 Australia; 2 Department of Agriculture and Food, Pork Innovation, South Perth, WA 6151 Australia; 3 John L Black Consulting, Warrimoo, NSW 2774 Australia; 4 SunPork Farms Solutions, Loganholme, QLD 4129 Australia; 5 Barneveld Nutrition Pty Ltd, Loganholme, QLD 4129 Australia; 6 Present address: AB Vista Asia Pte. Ltd., Balestier Road, The Mezzo, 329682 Singapore, Singapore

**Keywords:** Acetylsalicylic acid, *E. coli* infection, PGE_2_, Vitamin E, Weaner pigs

## Abstract

**Background:**

This experiment was conducted to test the hypothesis that vitamin E (Vit E) and acetylsalicylic acid (ASA), a cyclooxygenase-2 (COX-2) inhibitor, will additively reduce the production of the immunosuppressive molecule prostaglandin E_2_ (PGE_2_) and hence reduce inflammatory responses in weaner pigs experimentally infected with an enterotoxigenic strain of *E. coli*.

**Methods:**

The experiment was conducted in a research facility with 192 individually-housed male weaner pigs (Landrace × Large White) weighing 6.6 ± 0.04 kg (mean ± SEM). The pigs were experimentally infected with an enterotoxigenic strain of *E. coli* and were allocated to a 2 × 3 factorial design with the respective factors being without and with 125 ppm ASA and three levels of Vit E supplementation (50, 100 or 200 IU/kg diet, *dl*-α-tocopheryl acetate).

**Results:**

Acetylsalicylic acid supplementation improved average daily gain (*P <* 0.05) and tended to improve feed:gain ratio (*P <* 0.10) during the first 14 d after weaning. Acetylsalicylic acid supplementation also improved (*P* < 0.001) amino acid utilization efficiency (as assessed by plasma urea level) and tended to decrease (*P* < 0.10) PGE_2_ production in the liver without affecting small intestinal histology and tight junction protein mRNA expression in the jejunal epithelium. Vitamin E supplementation greater than 100 IU/kg diet sustained both the plasma Vit E concentration (*P* < 0.001) and plasma haptoglobin content (*P* < 0.001) after weaning. However, there was no additive effects of the combined supplementation of ASA and Vit E on performance, intestinal barrier function and inflammatory responses of weaned pigs.

**Conclusions:**

Although ASA and vitamin E improved amino acid utilization efficiency and reduced acute inflammatory responses, ASA and vitamin E did not additively reduce production of PGE_2_ and inflammatory responses in weaner pigs experimentally infected with an enterotoxigenic strain of *E. coli*.

## Background

Immune system activation alters nutrient partitioning from deposition of body protein to production of immune molecules [1] and provokes inflammatory responses such as fever, inhibition of appetite and vomiting, which compromise the wellbeing and welfare of pigs [2, 3]. These responses are caused by release of pro-inflammatory cytokines and subsequent synthesis of the immunosuppressive molecule prostaglandin E_2_ (PGE_2_), which is synthesized from arachidonic acid in many tissues [4]. Therefore, reducing PGE_2_ production by blocking conversion of arachidonic acid to PGE_2_, which is facilitated by cyclooxygenase-2 (COX-2), will reduce inflammatory responses and may have positive effects on the health, welfare and performance of weaner pigs [4]. Previously, acetylsalicylic acid (ASA**,** 125 ppm, inhibiting COX-1 and COX-2) in diets for weaner pigs was reported to improve performance and reduced diarrhea [5].

Another ingredient that has the ability to block PGE_2_ biosynthesis through prevention of lipid peroxidation is vitamin E (Vit E) [6]. Weaning and *E. coli* infection are known to reduce body Vit E reserves [7, 8], suggesting a possible association between Vit E and inflammatory responses. This significant reduction in whole body Vit E reserves in the first few wk after weaning is most likely caused by an increased requirement for immune function during this critical period. Likoff et al. [9] demonstrated a significant additive effect of Vit E (300 IU/kg diet) and ASA (intraperitoneal injection of 50 mg/kg body weight) supplementation on depression of PGE_2_ production and mortality in *E. coli* (LD_50_)-infected broilers. However such a study to examine the additive effects of ASA and Vit E supplementation has not been conducted in weaner pigs using experimental infection with an enterotoxigenic strain of *E. coli*. Therefore, this experiment examined the hypothesis that supplementation of Vit E and ASA, a COX-2 inhibitor, will additively reduce production of PGE_2_ and hence reduce the inflammatory responses in weaner pigs experimentally infected with an enterotoxigenic strain of *E. coli*.

## Methods

The experimental protocol used in this study was approved by the Department of Agriculture and Food Western Australia Animal Ethics Committee (AEC 6-12-19). Animals were handled according to the Australian Code of Practice for the Care and Use of Animals for Scientific Purposes [10].

This experiment was conducted to examine the individual and additive effects of ASA and Vit E on growth performance, aspects of gastrointestinal tract (GIT) structure and function, inflammation responses measured as white blood cell count and haptoglobin content, and PGE_2_ biosynthesis in the liver and spleen.

A total of 192 individually-housed male weaner pigs (Landrace × Large White) weighing 6.6 ± 0.04 kg (mean ± SEM) were allocated to a 2 × 3 factorial design, without and with 125 ppm ASA (acetylsalicylic acid; Bayer Australia, Pymble, NSW) and three levels of Vit E supplementation (50, 100 or 200 IU *dl*-α-tocopheryl acetate/kg diet; DSM, Wagga Wagga, Australia). This study mimicked the infection pressure of commercially housed pigs at weaning using an experimental *E. coli* infection model based on the assumption that *E. coli* infection with F4 fimbria after weaning remains a major cause of GIT dysfunction in some parts of the world [11–17]. There were 32 replications per treatment combination, with the experiment conducted in two consecutive batches of 96 pigs. A wheat, soybean meal and skim milk powder-based basal diet was formulated to contain 15.3 MJ digestible energy (DE)/kg (10.7 MJ net energy/kg) and 0.9 g standardized ileal digestible lysine/MJ DE (Tables 1 and 2).

**Table 1:** Ingredients and calculated composition of the basal diet (as-fed basis)^a^

Ingredients	%, as-fed
Barley	10.0
Wheat	51.33
Canola meal	1.24
Soybean meal	10.0
Full fat soya	5.0
Blood meal	1.24
Fishmeal	4.72
Skim milk powder	10.0
Canola oil	3.88
L-Lysine	0.405
DL-Methionine	0.24
L-Threonine	0.164
L-Tryptophan	0.01
Vit/Mineral premix^b^	0.07
Limestone	0.75
Dicalcium phosphate	0.75
Salt	0.20
Calculated composition	
DE, MJ/kg	15.3
Crude protein, %	21.0
SID lysine, g/MJ DE	0.9

^a^Acetylsalyacyl acid and vitamin E were replaced with wheat on a weight basis in the respective diets

^b^BJ Grower 1 (BioJohn Pty Ltd, Belmont, WA, Australia), provided the following nutrients (per kg of air-dry diet): vitamins: A 7,000 IU, D3 1,400 IU, E 20 mg, K 1 mg, thiamine 1 mg, riboflavin 3 mg, pyridoxine 1.5 mg, cyanocobalamin 15 μg, calcium pantothenate 10.7 mg, folic acid 0.2 mg, niacin 12 mg, biotin 30 μg. Minerals: Co 0.2 mg (as cobalt sulfate), Cu 10 mg (as copper sulfate), I 0.5 mg (as potassium iodine), Fe 60 mg (as ferrous sulfate), Mn 40 mg (as manganous oxide), Se 0.3 mg (as sodium selenite), Zn 100 mg (as ZnO)

**Table 2:** Analyzed amino acid and vitamin E contents of the experimental diets (as-fed basis)

Acetylsalicylic acid	0 ppm			125 ppm		
Added Vitamin E/kg diet	50 IU	100 IU	200 IU	50 IU	100 IU	200 IU
Amino acids, % as-fed						
Indispensable amino acids						
His	0.65	0.58	0.59	0.60	0.59	0.60
Ile	0.90	0.85	0.85	0.87	0.87	0.88
Leu	1.80	1.68	1.69	1.71	1.71	1.73
Lys	1.51	1.44	1.58	1.45	1.46	1.43
Met	0.72	0.66	0.67	0.68	0.71	0.67
Phe	1.14	1.04	1.06	1.08	1.08	1.09
Thr	1.19	1.06	1.09	1.04	1.06	1.11
Val	1.10	1.02	1.01	1.03	1.03	1.02
Dispensable amino acids						
Ala	1.01	0.94	0.95	0.96	0.96	0.97
Arg	1.27	1.18	1.19	1.20	1.20	1.21
Asp	2.01	1.85	1.89	1.88	1.89	1.94
Cys	0.42	0.37	0.42	0.42	0.41	0.44
Glu	4.98	4.48	4.68	4.72	4.69	4.78
Gly	0.99	0.95	0.96	0.95	0.94	0.96
Pro	1.93	1.93	1.82	1.92	1.79	1.68
Ser	1.26	1.14	1.17	1.16	1.17	1.21
Tyr	0.83	0.78	0.80	0.79	0.80	0.80
α-tocopherol, mg/kg as-fed	36	111	171	31	102	161

Pigs were fed experimental diets *ad libitum* and fresh water was supplied through a bowl drinker. All pigs were orally infected with an enterotoxigenic strain of *E. coli* (ETEC; serogroup O149:K91:F4) on d 7, 8 and 9 after weaning through oral drenching of 6, 10 and 10 mL of ETEC solution containing 1.5 × 10^8^ CFU/mL, respectively. The *E. coli* solution was freshly prepared on the day of dosing according to the method described in Heo et al. [18]. On d 10 after weaning, eight selected pigs (close to median weight) per treatment were euthanized and the remaining 24 pigs were used for performance measurement for a total duration of 21 d. Pigs were weighed and feed intake was recorded weekly to calculate performance indices. Expression of diarrhea and the number of antibiotic treatments were recorded daily for 14 d after weaning. Blood samples were collected in lithium-heparin tubes from all pigs on d 0, 7, 14 and 21 to determine the treatment effect on plasma vitamin E, plasma urea, and haptoglobin over time. Additional blood samples were collected in EDTA tubes from euthanized pigs on d 10 to measure whole blood immune cell counts.

Pigs were monitored daily for the presence of diarrhea. Feces were scored daily depending on their consistency using the following criteria: 1 = well-formed feces, firm to cut; 2 = formed feces, soft to cut; 3 = feces falling out of shape upon contact with surfaces and sloppy; 4 = pasty and liquid diarrhea. Piglets were counted as having diarrhea when the fecal consistency score was 4. To comply with Animal Ethics Committee requirements, pigs with diarrhea were treated immediately with an intramuscular injection of Trisprim-480 (trimethropin 80 mg/mL, sulfadiazine, 400 mg/mL; Troy Laboratories, Smithfield, Australia) or Moxylan (amoxicillin 150 mg/mL, Jurox Pty Ltd., Rutherford, Australia), and this was repeated daily until the diarrhea ceased. The diarrhea index was then expressed as the proportion of days with diarrhea over 14 d after weaning. The number of therapeutic antibiotic treatments was recorded for the first 14 d. Fecal β-hemolytic *E. coli* shedding was measured on d 0, 7, 9, 11 and 13, by gently swabbing the rectum with a cotton bud and overnight incubation of a fecal swab at 37 °C using 5 % horse blood agar plates [19].

### Postmortem procedures

The eight median weight pigs per treatment were euthanized on d 10 after weaning. Pigs were administered a single intramuscular injection of 2 mg Xylazine/kg (10 mg xylazil, Ilium Xylazil-100, Troy Laboratories Pty Ltd, Smithfield, Australia) and 5 mg Zoletil/kg body weight (10 mg tiletamine + 10 mg zolazepam, Zoletil 100, Virbac Pty Ltd, Peakhurst, Australia) to induce general anesthesia, and then euthanized by intracardiac injection of a lethal dose (2 mL/kg) of sodium pentobarbitone solution (Lethabarb; 325 g/mL pentobarbitone sodium, Virbac Australia Pty Ltd, Peakhurst, Australia). The abdomen was then immediately opened from the sternum to the pubis, and the GIT, liver and spleen were removed. The small intestine was stripped free of its mesentery and placed on a table into sections of equal length. For measurement of villous height and crypt depth, 3–4-cm segments of the small intestine were removed at the jejunum (approximately midway along the small intestine) and ileum (5 cm cranial to the ileo-cecal junction), carefully washed with phosphate-buffered saline (PBS) and preserved in 10 % phosphate-buffered formalin solution for subsequent histological examination. Approximately 5 g of tissue from the right lobe of the liver and anterior end of the spleen were collected, washed with PBS, snap frozen in liquid nitrogen and stored at −80 °C for subsequent analyses of PGE_2_ and COX-2. Mucosal samples for tight junction protein gene expression analysis were collected from approximately 20 cm-segments of the mid-jejunal region of the small intestine. The jejunum was opened, washed with PBS, collected mucosal scrapings using a sterile surgical blade, and stored in RNA stabilizer to prevent RNA degradation (RNAlater, Qiagen, Chadstone, Australia). The sample was stored at 4 °C for 24 h and then stored at −20 °C until required for DNA extraction.

### Chemical analysis

Amino acid (AA) contents in the experimental diets were measured according to a method described by Ranyer [20] with the modification of Barkholt and Jensen [21]. Briefly, a 100 mg sample was hydrolysed with 6 mol/L HCl, 0.5 % phenol and 0.05 % dithiodipropionic acid to convert protein-bound AA to free AA. The AA in the hydrolysate then underwent pre-column derivatization with o-phthalaldehyde and fluorenylmethylchloroformate according to Hewlett Packard Technical Note PN 12-5966-311E. The AA derivatives were then separated and quantified by Reverse Phase HPLC (Hewlett Packard 1100 HPLC with Diode Array Detector). An Aglient Hypersill AA-ODS column (200 mm × 2.1 mm, 5 microns) with pre-column was used for all analyses. The column temperature employed was 30 °C, detection was at 338 nm for primary AA (all amino acids detected at 338 nm except proline which is detected at 265 nm) and 262 nm for secondary AAs, and the flow rate was 0.3 mL/min.

Vitamin E acetate (α-tocopherol) content in the feed was measured using the method of McMurray et al. [22]. Briefly, 1 g of feed was homogenised in 10 mL of 6 % pryrogallol by Ultraturrex. One mL of 60 % KOH in water was added and the sealed tubes were heated at 70 °C for 30 min. After cooling, 5 mL of water and 20 mL of hexane was added. After extraction by vortexing, 5 mL of the hexane layer was evaporated under nitrogen and made up in 0.5 mL of methanol (0.1 % butylated hydroxytoluene). The chromatographic separation was performed with an Agilent HPLC system (1100) using a Zorbax SB-C18 column (3 mm × 150 mm, 3.5 μm, Agilent). Alpha-tocopherol was quantified using Fluorescence Detection (excitation 296 nm and emission 330 nm).

Alpha tocopherol content in the plasma sample was analysed using the method of McMurray and Blanchflower [23]. Briefly, 1 mL of plasma was deproteinized with 1 mL of 1 % pryrogallol in ethanol and 5 mL of hexane was added. After extraction by vortexing, 4 mL of the hexane layer was evaporated under nitrogen and made up in 0.5 mL of methanol (0.1 % butylated hydroxytoluene). The chromatographic separation was performed with an Agilent HPLC system (1100) using a Zorbax SB-C18 column (3 mm × 150 mm, 3.5 μm) (Agilent) with a methanol mobile phase. Alpha-tocopherol was quantified using fluorescence detection (excitation 296 nm and emission 330 nm).

Haptoglobin content in the plasma sample was determined using a modified method of Makimura and Suzuki [24]. Modifications were first, a higher concentration of sodium dihydrogen phosphate dihydrate (30 mmol/L in reaction mix), and second, the use of a commercial supply of hemoglobin (Sigma-Aldrich, H2625) to produce the hemoglobin reagent (30 g/L in normal saline). The method was adapted onto an Olympus Au400 Automatic Analyzer (Olympus, Tokyo, Japan).

Plasma urea content was measured using a urease kinetic method with an automatic analyser (Randox Daytona, Crumlin Co., Antrim, UK). Cyclooxygenase-2 (Novatein Biosciences, Cambridge, USA) and PGE_2_ (R&D Systems Inc., Minneapolis, USA) contents in the liver and spleen samples were measured using commercially available enzyme-linked immunosorbent assay (ELISA) kits. Whole blood immune cell count was done using an Automatic Hematology Analyzer (ADIVA 2120, Bayer Healthcare, Siemens, Germany).

For mucosal histology examination, ring-shaped sections of the intestine from the jejunum and ileum were excised, dehydrated, and embedded in paraffin wax, after fixation for several days in 10 % phosphate-buffered formalin. From each of these, six transverse sections (4–6 μm) were cut, stained with hematoxylin and eosin, and mounted on glass slides. The height of 10 well oriented villi, their associated crypts, and thickness of the muscular layer were measured with a light microscope (OLYMPUS CX31, Tokyo, Japan) using a calibrated eyepiece graticule.

Expressions of the mRNA encoding tight junction proteins, Zonula Occludin-1 (ZO-1) and occludin, in the jejunal mucosal scrapings were determined by a reverse transcription-polymerase chain reaction (RT-PCR). For RNA extraction, approximately 100 mg of mucosal tissue scraping from the jejunum was placed into 1 mL of TRIzol Reagent (Invitrogen, Mt Waverly, Australia). This was then homogenized using a tissue homogenizer for 45 s. Total RNA was extracted using the PureLink RNA mini kit (Invitrogen, Mt Waverly, Australia) according to the manufacturer’s instructions. Any possible contamination of genomic DNA was eliminated using PureLink DNase treatment (Invitrogen, Mt Waverly, Australia). The RNA was reverse transcribed in a 50 μL final volume using Superscript III (RT-SSIII) reverse transcriptase (100 IU, Invitrogen, Waltham, USA) in 5 × RT buffer, with 2.5 ng/μL random primers, 10 mmol/L each deoxynucleoside-triphosphate, 0.1 mol/L dithiotreitol and 20 IU RNAsin. A heat start was applied for 2 min at 50 °C and then the RT-SSIII was added. Samples were incubated at 45 °C for 50 min and then 55 °C for 30 min. The RT enzyme was heat inactivated (90 °C for 5 min). Real-time PCR was performed using a Corbett Rotor-Gene 3000 Real Time Thermal Cycler (Corbett Research, Sydney, Australia). The reactions were performed in the presence of conventional forward and reverse primers and SYBR Green (Invitrogen, Mt Waverly, Australia). Primers for the target genes’ occludin and ZO-1 used are presented in Table 3. Expression of occludin and ZO-1 were normalized to an endogenous control gene (Actin, β) to give a ΔCt value. This accounted for variability in the initial starting amount of cDNA. An aliquot of a previously run sample from a standard curve with a known Ct value was also placed in every run, to compare run-to-run variance and to determine the amount of the gene. Cycling conditions for RT-PCR consisted of two holds of 50 °C for 2 min, 95 °C for 10 min and then cycling for 40 cycles for 95 °C 15 s, 60 °C for 1 min and 60 °C for 60 s. Each sample was run in triplicate. The Pfaffl [25] method was used to calculate the relative expression of a target gene based on the efficiency of the primers used and the Ct deviation of the unknown gene versus a control, and expressed in comparison to a reference gene.

**Table 3:** Primer sequences and conditions used for real-time PCR

		Product	Sequence	Concentration
Primer	Sequence	size, bp	GenBank ID	nmol/L^a^
Occludin				
Forward	5′-GCAGCAGTGGTAACTTGGA-3′	113	NM_001163647.2	200
Reverse	5′-GTCGTGTAGTCTGTCTCGTAATG-3′			
ZO-1				
Forward	5′-CGGCGAAGGTAATTCAGTGT-3′	109	XM_003353439.2	200
Reverse	5′-CGGTTTGGTGGTCTGTAAGT-3′			
Actin β				
Forward	5′-GCCCGTCCATCGTCCACCG-3′	127	XM_003357928	200
Reverse	5′-CAGGAGGCTGGCATGAGGTGTG-3′			

^a^Final concentration of primers used in real-time PCR

### Statistical analysis

Data were analyzed by two-way ANOVA using the pig as the experimental unit. The main and interactive effects of the supplemental Vit E and ASA were included as fixed factors in the statistical model. As there was a significant batch effect, it was included as a random factor in the statistical model. Plasma urea, haptoglobin and Vit E contents measured at the start of experiment (0 d) were used as covariates for statistical analysis on subsequent measurement days. Fisher’s protected-LSD test was used to separate means with significant treatment effects. Pearson’s correlation analysis was conducted to detect relationships between dietary treatments, production of COX-2 and PGE_2_ in the liver, expression of tight junction protein genes in the jejunal epithelium, and proportion of white blood cells. Variability in the data was expressed as the SEM. A probability level of *P* < 0.05 was considered to be statistically significant and *P* < 0.10 was considered as a trend. All statistical analysis was conducted using Genstat 15^th^ edition (VSN International Ltd, Hemel Hempstead, UK).

## Results

### Performance and indices of post-weaning colibacillosis

Supplementation of ASA alone improved (*P* < 0.05) average daily gain (ADG) and tended to improve (*P* < 0.10) the gain:feed (G:F) ratio during the first 14 d after weaning (Table 4). Significant interactions (*P* < 0.05) occurred between ASA and Vit E for indices of post-weaning colibacillosis, namely the diarrhea index and the number of antibiotic treatments, such that supplementation of 200 IU Vit E/kg diet decreased the diarrhea index and the number of antibiotic treatments but only with concurrent supplementation of 125 ppm ASA. Supplementation of ASA and 100 IU Vit E/kg diet independently decreased (*P* < 0.05) the fecal β-hemolytic *E. coli* score (Table 4).

**Table 4:** The effects of acetylsalicylic acid (ASA) and vitamin E (Vit E) on performance and indices of post-weaning colibacillosis measured for 21 d after weaning in *E. coli*-infected pigs^a^

	ASA (A), ppm			Vit E (E), IU				*P*-value		
	0	125	SEM	50	100	200	SEM	A	E	A × E
D 0 – 14										
ADG, g/d	154	173	5.4	175	163	152	5.4	0.034	0.119	0.375
ADFI, g/d	180	182	5.2	190	181	171	5.2	0.998	0.090	0.503
G:F	0.87	0.94	0.022	0.93	0.90	0.88	0.022	0.087	0.587	0.175
D 0 – 21										
ADG, g/d	234	245	5.6	250	240	230	5.6	0.218	0.183	0.614
ADFI, g/d	279	288	6.4	290	280	280	6.4	0.469	0.678	0.867
G:F	0.86	0.87	0.013	0.87	0.86	0.86	0.013	0.742	0.894	0.412
Diarrhoea index, %^b^	8.2	6.0	0.78	7.1	6.4	7.8	0.79	0.126	0.701	0.043
Antibiotic^c^	1.8	1.7	0.16	1.8	1.8	1.7	0.17	0.724	0.910	0.023
*E. coli* score^d^	8.0	6.3	0.79	7.4	5.8	8.2	0.79	0.009	0.002	0.391

^a^All pigs were experimentally infected with an enterotoxigenic strain of *E. coli* on 7, 8 and 9 d after weaning. The numbers of replications were 32 individual pigs up to d 10 and 24 pigs from d 14 to 21

^b^Diarrhea index, %; mean percentage of days with diarrhea with respect to first 14 d after weaning. Significant interaction (*P* < 0.05) occurred between ASA and Vit E for the diarrhoea index, such that supplementation of 200 IU Vit E/kg diet decreased diarrhoea index but only with concurrent supplementation of 125 ppm ASA

^c^Mean number of antibiotic treatments. Significant interaction (*P* < 0.05) occurred between ASA and Vit E for the number of antibiotic treatments, such that supplementation of 200 IU Vit E/kg diet decreased the number of antibiotic treatments but only with concurrent supplementation of 125 ppm ASA

^d^Mean cumulative *E. coli* scores per diet in the 14 d after weaning

### Plasma Vit E, urea, haptoglobin levels, and blood cell counts

**Fig. 1:**
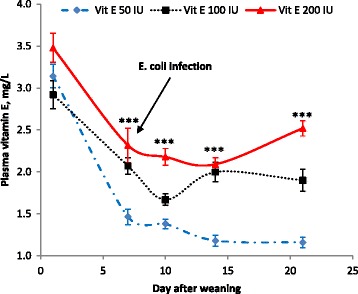
Effect of dietary vitamin E levels on plasma vitamin E contents after weaning. The numbers of replications were 32 individual pigs up to d 10 and were 24 pigs from d 14 to 21. All pigs were experimentally infected with an enterotoxigenic strain of *E. coli* on d 7 (after blood sampling), 8 and 9. ***P < 0.001

**Fig. 2:**
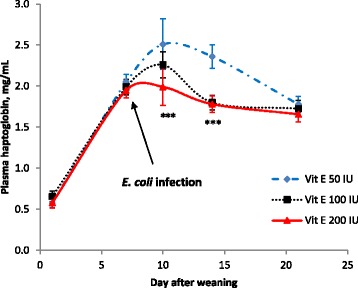
Effect of dietary vitamin E levels on plasma haptoglobin content after weaning. The numbers of replications were 32 individual pigs up to day 10 and were 24 pigs from day 14 to 21. All pigs were experimentally infected with an enterotoxigenic strain of *E. coli* on day 7 (after blood sampling), 8 and 9. *P < 0.05, ***P < 0.001

Increasing the dietary Vit E content decreased (*P* < 0.001) the extent of the reduction of plasma Vit E content after weaning on all measured days (Fig. 1). Increasing Vit E supplementation from 50 IU to 100 IU or 200 IU/kg diet decreased plasma haptoglobin content after *E. coli* infection (2.51, 2.26 and 1.99 mg/mL, respectively on d 10, *P* < 0.05; 2.36, 1.8 and 1.78 mg/L, respectively on d 14, *P* < 0.001, Fig. 2), while ASA supplementation had no effect (*P* > 0.05) on plasma haptoglobin content (2.31 and 2.20 mg/L on d 10; 2.05 and 1.91 mg/L on d 14 for without and with ASA, respectively).

**Fig. 3:**
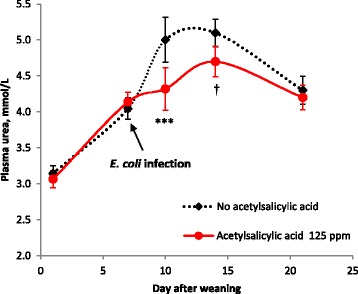
Effect of acetylsalicylic acid (125 ppm) on plasma urea contents after weaning. The numbers of replications were 32 individual pigs up to d 10 and were 24 pigs from d 14 to 21. All pigs were experimentally infected with an enterotoxigenic strain of *E. coli* on d 7 (after blood sampling), 8 and 9. ***P < 0.001, †*P* < 0.10

Acetylsalicylic acid supplementation decreased plasma urea content after oral *E. coli* infection (5.0 vs. 4.3 mmol/L on d 10, *P* < 0.001; 5.1 vs. 4.7 mmol/L on d 14, *P* = 0.072) (Fig. 3), while Vit E supplementation had no significant effect on plasma urea content (4.8, 4.7 and 4.5 mmol/L on d 10; 4.8, 4.8 and 5.0 mmol/L on d 14 for 50, 100 and 200 IU Vit E/kg, respectively). Increasing the dietary Vit E content from 50 to 100 and 200 IU/kg diet increased the number of platelets without ASA supplementation (447, 488 and 569 × 10^9^/L, respectively), but decreased the number of platelets with ASA supplementation (549, 598 and 435 × 10^9^/L, respectively; interaction *P* = 0.058). Regardless of ASA supplementation, Vit E supplementation at 100 IU increased (*P* < 0.05) white blood cell numbers, and tended to increase (*P* = 0.078) the proportion of neutrophils while tending to decrease (*P* = 0.093) the proportion of lymphocytes (Table 5).

**Table 5:** Effect of supplementation of acetylsalicylic acid (ASA) and vitamin E (Vit E) on selected blood cell numbers (*n* = 8) measured on d 10 after weaning after experimental infection with an enterotoxigenic strain of *E. coli*

Items	ASA (A), ppm			Vit E (E), IU				*P=*		
	0	125	SEM	50	100	200	SEM	A	E	A × E
RBC, × 10^12^/L	6.4	6.5	0.07	6.5	6.5	6.3	0.07	0.826	0.374	0.902
CHCM, g/L	270	274	1.7	271	271	274	1.7	0.077	0.428	0.203
Platelet, × 10^9^/L	501	527	24.5	498	543	502	24.7	0.575	0.675	0.058
WBC, × 10^9^/L	17.6	19.2	1.00	16.7	22.3	16.2	0.93	0.407	0.017	0.938
Neutrophil, %^a^	48.9	49.7	1.63	46.8	54.2	46.9	1.57	0.769	0.078	0.576
Lymphocyte, %^a^	44.9	44.4	1.60	47.1	40.0	46.8	1.54	0.856	0.093	0.675

*RBC* red blood cell, *CHCM* mean red blood cell hemoglobin content, *WBC* white blood cell

^a^Expressed as a percentage of total white blood cell count

### Small intestinal structure, tight junction protein mRNA expression in the jejunum, and PGE_2_ and COX-2 concentrations in the liver and spleen

There was a trend for supplementation of Vit E to increase (*P* = 0.089) crypt depth in the ileum (202, 260, 223 μm for 50, 100 and 200 IU Vit E/kg diet, respectively), otherwise ASA and Vit E supplementation had no significant effects on small intestinal structure (results not shown).

Relative expression of mRNA for tight junction proteins, occludin and ZO-1, were measured in the jejunal epithelium. There was large within-treatment variation in mRNA expression for tight junction proteins that limited detection of statistical differences between treatments.

**Fig. 4:**
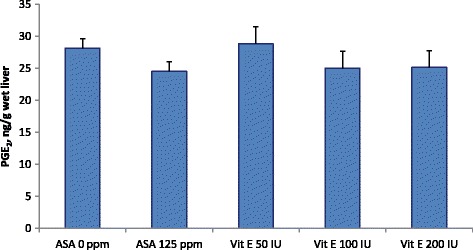
Effect of an acetylsalicylic acid (125 ppm) and dietary vitamin E levels on PGE_2_ concentration in the liver measured after 24 h of experimental infection with an enterotoxigenic strain of *E. coli* in weaner pigs. The numbers of replications were 8 individual pigs. All pigs were experimentally infected with an enterotoxigenic strain of *E. coli* on d 7, 8 and 9. Effect of acetylsalicylic acid *P =* 0.085; Vitamin E effect was not significant

Acetylsalicylic acid tended to decrease PGE_2_ production in the liver (24.5 vs. 28.2 ng/g wet tissue, *P* = 0.085, Fig. 4). However, COX-2 contents in the liver were low and showed no statistical differences between treatments.

### Relationships between treatments and measured variables

Acetylsalicylic acid intake was negatively correlated to PGE_2_ content in the liver (*P* < 0.05). COX-2 concentration in the liver was negatively correlated to tight junction protein gene expression in the jejunal epithelium (occludin *P* < 0.05 and ZO-1 *P* < 0.001). Prostaglandin E_2_ concentration in the liver tended to positively correlate with the proportion of lymphocytes and neutrophils (*P* < 0.10) (Table 6).

**Table 6:** Pearson’s correlation coefficients between treatments and measured variables (Experiment 1)^a^

	Acetylsalicylic acid intake^b^	Vitamin E intake^a^	Liver COX-2	Liver PGE_2_	Occludin	ZO-1	%Lymphocyte
Vitamin E intake	−0.090						
Liver COX-2	0.061	−0.096					
Liver PGE_2_	−0.297*	−0.195	−0.141				
Occludin	0.037	−0.070	−0.357*	0.219			
ZO-1	−0.059	0.062	−0.460***	−0.092	0.422**		
%Lymphocyte^c^	0.026	0.047	−0.158	0.263†	0.231	0.062	
%Neutrophil^c^	−0.012	−0.052	0.137	0.278†	0.210	−0.031	−0.989***

Significance †*P* < 0.10, *P < 0.05, **P < 0.01, ***P < 0.001

^a^Correlation coefficient was derived from 48 observations (eight pigs per treatment × 6 treatments)

^b^Acetylsalicylic acid and vitamin E intakes were calculated by multiplying individual daily feed intake and dietary concentration of acetylsalicylic acid and vitamin E

^c^Percentage of the total WBC count

## Discussion

This study tested the hypothesis that Vit E supplementation and ASA, a COX-2 inhibitor, would additively reduce the production of PGE_2_ and hence reduce the inflammatory responses in weaner pigs experimentally infected with ETEC in a research facility.

Several on farm studies reported a strong negative relationship between weaning and Vit E reserves in the body [7, 26]. Prevention of lipid peroxidation via increasing whole body Vit E reserve has been suggested to reduce the synthesis of eicosanoid mediators via negative feedback mechanisms [4]. Therefore, one of the major interests of this experiment was to examine whether supplemental Vit E can increase Vit E body reserves after weaning and inflammation challenge, and whether the increased Vit E body reserve is associated with a reduction in PGE_2_ biosynthesis. Previous research showed that plasma Vit E concentrations decrease markedly, to less than 1.5 mg/L, when pigs were fed a diet containing less than 100 IU Vit E/kg diet after weaning [7] or infected with ETEC [8]. Considering the anti-oxidative properties of Vit E, its requirement would be expected to be greater when oxidative tissue damage is increased in the immediate post-weaning period, due either to stress and (or) (sub)clinical infection as a result of the significant pathogen loads in commercial production systems. Results in this study indicated that 200 IU Vit E/kg diet was required to maintain plasma Vit E levels to above 2 mg/L, which is in agreement with a previous report [26]. Although supplementation of 200 IU Vit E/kg diet was insufficient to maintain a plasma Vit E level of 3 mg/L, which has been suggested as the level required for optimum immune function [27], supplementation with 200 IU Vit E/kg diet maintained the plasma Vit E level to greater than 2 mg/L even after ETEC infection, and was also able to return the plasma Vit E level to 2.5 mg/L by d 21 after weaning. In this regard, Wilburn et al. [26] reported that a plasma Vit E level of 3 mg/L was not sustainable by supplementation of 300 IU Vit E alone, but was able to be achieved by concurrent supplementation of 300 IU Vit E/kg in the feed and 100 IU Vit E/L in the drinking water.

One pathway through which acute immune system activation depresses growth performance of pigs is stimulation of the central nervous system by eicosanoid mediators such as PGE_2_ [28, 29]. Recognition of pathogens and (or) stimulation of mast cells increases production of eicosanoid mediators via pro-inflammatory cytokines, which are causative for fever due to increased metabolic rate and anorexia due to reduced appetite [2, 4]. Production of eicosanoid mediators in the cell and nuclear membranes is initiated through the action of phospholipase A_2_ or C, which respectively convert phospholipid and diacylglycerol to arachidonic acid. Arachidonic acid is then converted to PGE_2_ by COX-2 [28, 29]. Unlike steroids and non-steroidal anti-inflammatory drugs (NSAIDs), manipulation of dietary nutrients may not directly inhibit the activities of phospholipase or COX-2. However, it was anticipated that enhancing intestinal barrier function and protection of cell damage through supplementation of Vit E would reduce production of eicosanoid mediators via negative feedback mechanisms and therefore would improve the health and growth efficiency of pigs [4, 30]. The results of the present study showed that Vit E supplementation significantly decreased the production of haptoglobin, an acute-phase protein, after ETEC infection and tended to increase the rate of cell proliferation in the ileum (increased crypt depth). Vitamin E supplementation (200 IU/kg) without ASA supplementation increased tight junction protein mRNA expression in the jejunal epithelium by 12–16 %. However a very large within-treatment variation (CV 45 %) hindered the generation of statistical significance. Moreover, Vit E supplementation did not influence COX-2 or PGE_2_ concentration in the liver, although again there was a numerical decrease (CV 27 %) in PGE_2_ concentration in pigs fed all Vit E-supplemented diets. These findings indicate that Vit E supplementation reduces the acute-phase protein response mainly by preventing oxidative cell damage under stress and ETEC infection, but has limited effect on preventing the progression of the inflammatory cascade via modulation of COX-2 activity and PGE_2_ production, which is inconsistent compared with findings in broilers [9].

Acetylsalicylic acid is known to directly inhibit COX-1 and COX-2 via acetylating serine residues in the active site of the enzymes, and hence eventually inhibits the production of PGE_2_ [31]. Acetylsalicylic acid is also known to interfere with blood clotting and wound healing by reducing thromboxane A_2_ in the platelets and inhibiting platelet aggregation [32]. Xu et al. [5] reported that adding 125-ppm ASA in a starter diet for weaner pigs decreased diarrhea and improved growth performance, whereas supplementation of 250 ppm ASA did not further improve performance and health. The results of the present study partly supported our hypothesis as ASA in the diet at 125 ppm tended (*P* = 0.085) to decrease PGE_2_ production in the liver without affecting small intestinal histology and tight junction protein mRNA expression in the jejunal epithelium. Pearson’s correlation analysis also confirmed that individual pig ASA intake was negatively correlated to PGE_2_ concentration in the liver. However, ASA supplementation did not significantly reduce COX-2 concentration in the liver (only numerical reduction), and this may be attributed to the ability of ASA to primarily inhibit COX-1 rather than COX-2 activity [31].

Weaning per se significantly increased plasma urea concentration from 3 mmol/L on the day of weaning to 4 mmol/L on d 7 after weaning, and experimental ETEC infection on d 7–9 further increased plasma urea concentration from 4 mmol/L to the level greater than 5 mmol/L on d 10 and 14 after weaning, representing metabolic waste of amino acids for the immune response [4]. However, pigs fed an ASA-supplemented diet maintained their plasma urea content to the pre-infection level, especially immediately after ETEC infection. These results most likely suggest that ASA supplementation improved amino acid utilization efficiency by inhibiting progression of inflammatory cascades through reducing biosynthesis of the immunosuppressive molecule PGE_2_. The finding that ASA supplementation improved ADG and tended to improve G:F only in the first 14 d after weaning may indicate that the metabolic waste of nutrients due to immune system activation is more severe during this period and then settled after 14 d, which is in agreement with the pattern of plasma urea content as demonstrated in the present study.

As the underlying mechanisms for Vit E and ASA on infection responses of pigs are different, an additive reduction of inflammatory response was hypothesized in pigs fed a diet supplemented with both Vit E and ASA. For example, it was reported that reducing bacterial load by supplementation of an antibiotic (doxycycline) and reducing PGE_2_ production by supplementation of ASA additively decreased rectal temperature in finisher pigs having respiratory disease [31]. Moreover, Likoff et al. [9] demonstrated a strong additive effect of Vit E (300 IU) and ASA (intraperitoneal injection of 50 mg/kg body weight) on depression of PGE_2_ production and mortality in *E. coli* (LD_50_)-infected broilers. However, such an additive effect was not observed in the present experiment except for interactions in the diarrhea index and antibiotic treatments, where concurrent supplementation of ASA and Vit E was more effective than individual supplementation alone. Based on the results of plasma and tissue measurements, therefore, it is likely that ASA and Vit E supplementation independently improved performance or inflammatory response of weaned pigs by reducing inflammation-associated amino acid waste through modulation of PGE_2_ biosynthesis, or by reducing the severity of infection through an eicosanoid-independent pathway such as oxidative tissue damage due to its antioxidant property, respectively.

## Conclusions

Individual and additive effects of ASA and Vit E supplementation on performance, GIT structure and function and aspects of immune function were investigated in ETEC-challenged weaned pigs. Acetylsalicylic acid and Vit E supplementation independently improved G:F and inflammatory response, respectively, but no additive effect was observed in performance, GIT structure and function or immune function of weaned pigs. Based on tissue measurements, it is suggested that ASA supplementation may have improved performance of weaned pigs by reducing inflammation-associated amino acid waste through modulation of PGE_2_ biosynthesis, while Vit E supplementation improved the inflammatory response after ETEC infection by reducing the severity of infection through an eicosanoid-independent pathway such as oxidative tissue damage, due to its antioxidant properties.

## Abbreviations

AA: Amino acid
ADFI: Average daily feed intake
ADG: Average daily gain
ASA: Acetylsalicylic acid
CFU: Colony forming unit
COX-2: Cyclooxygenase-2
DE: Digestible energy
ETEC: Enterotoxigenic *E. coli*
G:F: Gain to feed ratio
GIT: Gastrointestinal tract
PBS: Phosphate buffered saline
PGE_2_: Prostaglandin E_2_
Vit E: Vitamin E
ZO-1: Zonula occludin-1
